# Easy and quick (EQ) sperm freezing method for urgent preservation of mouse strains

**DOI:** 10.1038/s41598-021-93604-y

**Published:** 2021-07-08

**Authors:** Keiji Mochida, Ayumi Hasegawa, Daiki Shikata, Nobuhiko Itami, Masashi Hada, Naomi Watanabe, Toshiko Tomishima, Atsuo Ogura

**Affiliations:** 1 RIKEN BioResouce Research Center, Tsukuba, Ibaraki 305-0074 Japan; 2 Graduate School of Life and Environmental Science, University of Tsukuba, Tsukuba, Ibaraki 305- 8572 Japan; 3 Center for Disease Biology and Integrative Medicine, Faculty of Medicine, University of Tokyo, Bunkyo-ku, Tokyo, Japan; 4 RIKEN Cluster for Pioneering Research, Hirosawa, Wako, Saitama 351-0198 Japan

**Keywords:** Developmental biology, Biological techniques

## Abstract

Cryopreservation of mouse spermatozoa is widely used for the efficient preservation and safe transport of valuable mouse strains. However, the current cryopreservation method requires special containers (plastic straws), undefined chemicals (e.g., skim milk), liquid nitrogen, and expertise when handling sperm suspensions. Here, we report an easy and quick (EQ) sperm freezing method. The main procedure consists of only one step: dissecting a single cauda epididymis in a microtube containing 20% raffinose solution, which is then stored in a −80 °C freezer. The frozen–thawed spermatozoa retain practical fertilization rates after 1 (51%) or even 3 months (25%) with the C57BL/6 J strain, the most sensitive strain for sperm freezing. More than half of the embryos thus obtained developed into offspring after embryo transfer. Importantly, spermatozoa stored at −80 °C can be transferred into liquid nitrogen for indefinite storage. As far as we know, our EQ method is the easiest and quickest method for mouse sperm freezing and should be applicable in all laboratories without expertise in sperm cryopreservation. This technique can help avoid the loss of irreplaceable strains because of closure of animal rooms in emergency situations such as unexpected microbiological contamination or social emergencies such as the COVID-19 threat.

## Introduction

Cryopreservation of spermatozoa is an effective method for the preservation of mouse strains, especially gene-modified strains with a defined genetic background because their haploid genomes are sufficient for the propagation of modified genes based on particular background strains^1,2^. Importantly, a large number of spermatozoa can be obtained from a single male mouse, which enables the production of as many embryos as required using oocytes from wild-type females. The standard protocol for mouse sperm using a solution containing 3% skim milk and 18% raffinose was established 30 years ago^3^; since then, no major technical improvements have been made. This is because this original freezing protocol is applicable to spermatozoa from most major mouse strains^4^. Even spermatozoa from C57BL/6 J mice, which are highly sensitive to freezing and thawing^5–7^, maintain practical fertilizing ability after freeze–thawing, although the addition of methyl-β-cyclodextrin (MBCD)^8,9^ and reduced glutathione (GSH)^10,11^ is necessary . Thus, the current freezing protocol for mouse spermatozoa is practical enough for laboratories where sperm freezing is performed routinely. However, the protocol has some drawbacks that can hinder broader use in other laboratories where such cryopreservation is not routine. Drawbacks include the need for special containers (plastic straws), medium with a chemically undefined content (skim milk), and liquid nitrogen (LN_2_) for storage. Furthermore, some practice is necessary to acquire sufficient skill for handling sperm suspensions. Therefore, several minor attempts have been made to address these issues. Concerning the containers, we have devised a protocol for sperm freezing with conventional cryotubes instead of plastic straws^12^. Additionally, it was found that LN_2_ was not always necessary for the initial freezing and a few years of storage^13^. However, other issues remain unsolved. As far as we know, the simplest way of preserving male germ cells is freezing entire testes or male bodies in deep freezers or LN_2_^14^. Indeed, normal mice were born at high rates following intracytoplasmic sperm injection (ICSI) using spermatozoa retrieved from mouse bodies frozen for 15 years^14^. Furthermore, frozen testes can be transported by international couriers for supplying mice to other laboratories^14^. Thus, at least some of the spermatozoa frozen by these simple techniques can support full-term development. However, they need ICSI instead of in vitro fertilization (IVF) to generate embryos, because spermatozoa retrieved from these frozen tissues are completely immotile. Therefore, it is desirable to seek a simpler sperm freezing method than the current standard protocol, while maintaining the fertilizing ability of spermatozoa using conventional IVF.

Here, we aimed to solve the shortcomings of the current sperm freezing protocol so that inexperienced researchers can cryopreserve spermatozoa using existing or readily available materials. We have successfully devised a new method, which requires only conventional materials or devices such as 1.5-mL microtubes, raffinose, aluminum foil and a −80 °C deep freezer. Importantly, it does not require any special skills. We consider that our new sperm freezing protocol will be particularly effective in cases of immediate closure of animal rooms caused by accidental microbiological contamination or social emergencies such as the COVID-19 pandemic, which is relevant to every region of the world at present.

## Methods

### Animals

Females (11–15 weeks of age) and males (12–40 weeks of age) of the C57BL/6NJcl (B6N) and C57BL/6JJcl (B6J) mouse strains (CLEA Japan Inc., Tokyo, Japan) were used for sperm freezing and IVF experiments. Female mice of the ICR strain (CLEA Japan Inc.) at 10–20 weeks of age were used as recipients in embryo transfer experiments. All mice were maintained under a specific-pathogen-free condition, provided with water and commercial laboratory mouse chow ad libitum, and housed under controlled lighting conditions (daily light period, 07:00 to 21:00). All animal experiments described here were approved by the Institutional Animal Care and Use Committee of RIKEN Tsukuba Branch (no. T2020-004). All animal experiments were performed in accordance with Japanese legislation Act on Welfare and Management of Animals (Act No. 105, 1973; last version 2019) and the guiding principles of RIKEN Tsukuba Branch. All animal handling procedures complied with the ARRIVE guidelines for the Replacement, Refinement and Reduction of Animals in Research (NC3Rs) along the experimental period. At the time of collection of spermatozoa or oocytes, or of Cesarian section, animals were euthanized by cervical dislocation. For anesthesia of females during embryo transfer, 2.5% tribromoethanol (0.014 mL/g body weight) was administered by intraperitoneal injection.

### Sperm freezing by the standard method

Spermatozoa were frozen–thawed according to the method developed by Takeshima et al.^3^ with slight modifications. The sperm cryopreservation solution (R18S3) consisted of 18% raffinose (Difco, Becton Dickinson, Franklin Lakes, NJ, USA) and 3% skim milk (Difco). Fat and blood were removed from the epididymides on filter papers using fine forceps and scissors. Approximately 10 incisions were made into each cauda epididymis using fine scissors in 100-μL R18S3, and the resulting sperm suspension was divided into eight 10-μL aliquots. Each aliquot was aspirated into a 0.25-mL plastic straw (Cassou straw; IMV Technologies, L’Aigle Cedex, France) and cooled in LN_2_ vapor for 10–60 min before submersion in LN_2_. The spermatozoa were stored in LN_2_ for at least 1 week before thawing for motility analysis or IVF as described below.

We also performed sperm freezing experiments by the standard method to optimize the freezing solution for the EQ method. Spermatozoa were frozen in 15%, 18%, and 21% raffinose in different concentrations of supplemented Dulbecco’s (SD) phosphate-buffered saline (PBS)^15^ or distilled water (DW). After thawing, sperm motility was measured as described below.

### Kinetic analysis of fresh and frozen–thawed spermatozoa

Epididymal spermatozoa freshly collected or frozen–thawed were preincubated in human tubal fluid (HTF) medium^16^ for 1 h at 37 °C in 5% CO_2_ in humid air. The overall sperm motility, progressive motility, average path velocity (VAP), straight-line velocity (VSL), curvilinear velocity (VCL), amplitude of lateral head displacement (ALH), beat cross frequency (BCF), linearity (LIN), and straightness (STR) were assessed by computer-assisted sperm analysis using a Hamilton Thorn IVOS computerized semen analyzer (Hamilton Thorn, Beverly, MA, USA). Motile spermatozoa were defined as those with any movement of the sperm head. Spermatozoa with progressive motility were defined as those with forward, linear movement at a speed of > 50.0 μm/s. All these parameters were measured for ≥ 200 spermatozoa in at least three different microscope fields.

### Sperm freezing by the EQ method (Fig. 1)

**Figure 1:**
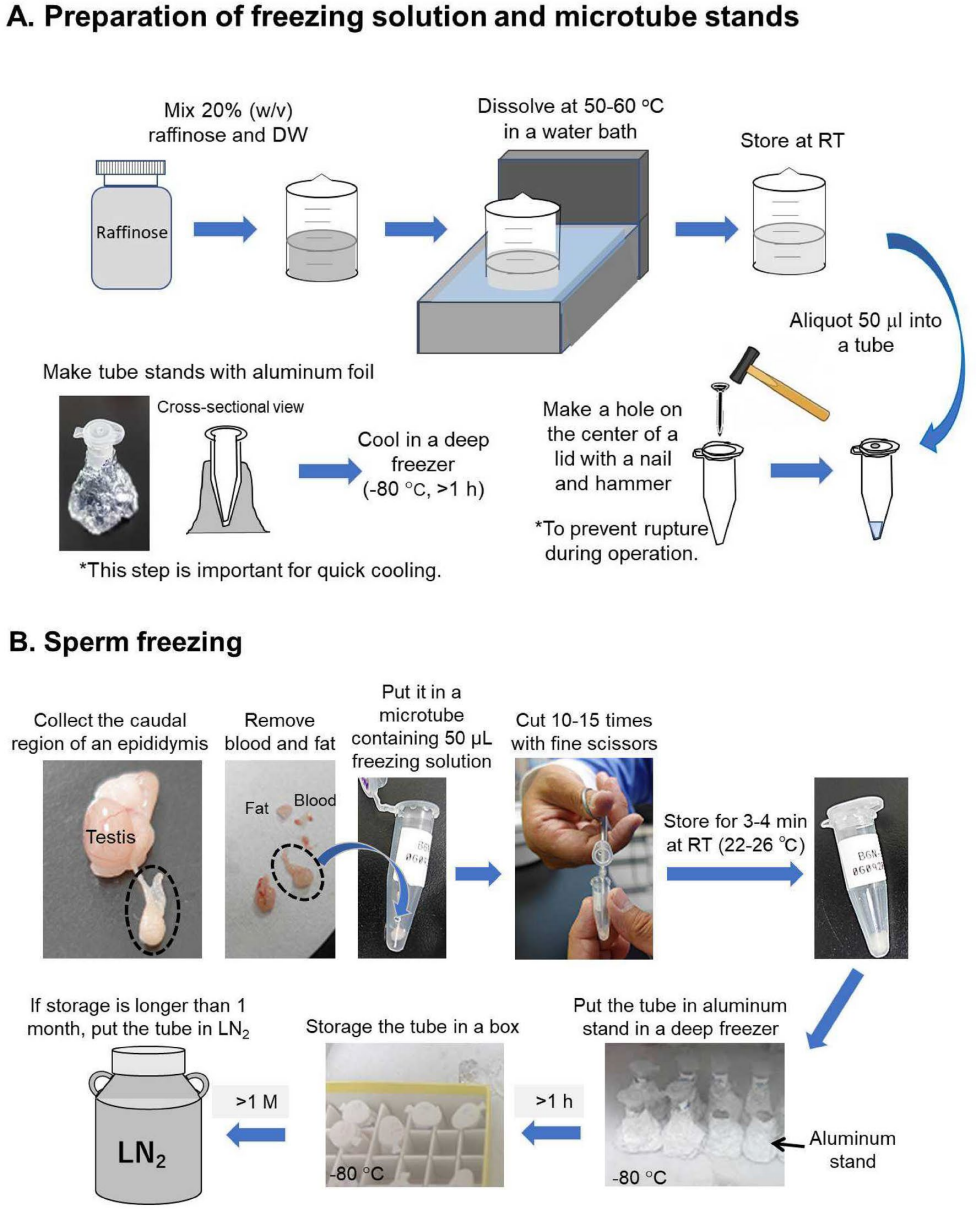
Schematic presentation of sperm freezing protocol by the EQ method.

First, a hole < 1 mm in diameter was made on the center of the lid of a 1.5-mL microtube by drilling with a nail and hammer (Fig. 1A). Use of LN_2_-resistant polypropylene microtubes is recommended. One microtube was prepared for each epididymis (two microtubes for each mouse). The microtube stands were constructed from aluminum foil by wrapping empty microtubes (Fig. 1A). Aluminum foil stands without microtubes were precooled in a deep freezer at −80 °C (or −40 °C in some experiments) at the upright position for more than 1 h. The cryoprotectant solution was prepared by dissolving 20% (w/v) raffinose in DW at 50–60 °C and stored at room temperature (Fig. 1A). Sperm suspensions for freezing were prepared in the following order. The caudal regions of epididymides collected from euthanized male mice were placed on filter papers to remove the blood and fat using fine forceps and scissors (Fig. 1B). Each was put on the bottom of a microtube containing 50 μL sperm freezing solution. Using fine sterile scissors, 10–15 incisions were made in the freezing solution (Fig. 1B). The lid was put on the microtube without removal of tissue residues, and this was then left to stand in an upright position at room temperature (22–25 °C) to allow spermatozoa to disperse. At 3–4 min later, each microtube was put into an aluminum foil stand in a deep freezer at –80 °C (or −40 °C for comparison). At least 1 h later, the microtubes were transferred to a freezer box and stored until use for kinetic analysis or the IVF experiments described below. It was important to make sure that there was no space between the aluminum foil and the microtube so that the sperm suspension could be cooled efficiently (see cross-sectional view in Fig. 1B).

## Measurement of cooling rates

The temperature was measured for each condition every 3 s using dummy microtubes containing the freezing solution inserted into the thermo probe (1 mm diameter: BS11E-005-TS1-ASP) attached to a Thermo Printer (AP-310, Anritsu Meter Co. Ltd, Tokyo, Japan). Then, the cooling rates were compared with previous data using a plastic straw and cryotube^12^.

### Superovulation

Female C57BL/6 mice for IVF were superovulated by hormone treatments; for this they were injected intraperitoneally with 7.5 IU equine chorionic gonadotropin (eCG; PMSA, Sankyo Co. Ltd., Tokyo, Japan) or anti-inhibin serum (100 μL per mouse; Central Research Co. Ltd, Tokyo, Japan)^17^, followed by injection with 7.5 IU human chorionic gonadotropin (hCG; Puberogen, Sankyo) 48 h later.

### Preparation of sperm preincubation and IVF media

**Figure 2:**
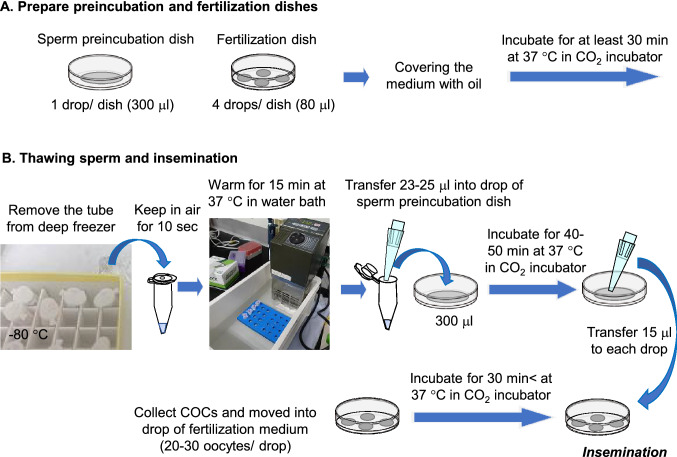
Schematic presentation of in vitro fertilization (IVF) using sperm frozen-thawed by the EQ method.

For the fertilization medium, HTF medium containing hypotaurine (0.11 mg/mL, Sigma-Aldrich, St Louis, MO, USA) and 0.3% bovine serum albumin (BSA; Calbiochem, Merck Millipore, Darmstadt, Germany) was supplemented with 1.25–1.5 mM GSH^10,12^. Then, four 80-μL droplets of the fertilization medium were prepared in a 35-mm plastic dish, covered with sterile mineral oil, and equilibrated in an incubator at 37 °C under 5% CO_2_ in humid air for at least 20 min (Fig. 2A). For the sperm preincubation medium, a 300-μL drop of HTF medium containing 0.4 mM MBCD^8,9^ and 0.1 mg/mL polyvinyl alcohol (PVA) instead of BSA were prepared in a dish, covered with sterile mineral oil, and incubated under the same conditions as the fertilization medium (Fig. 2A). In some experiments, 4.6 mg of CaCl_2_・2H_2_O were added to 10 mL of the sperm preincubation medium to increase the Ca^2+^ concentration to 5 mM.

### In vitro fertilization

This was performed as described with slight modifications^12,18^. In brief, 16–17 h after hCG injection, mature metaphase II (MII) oocytes were collected from the oviducts of females by puncturing the ampulla with a 26–27 G needle in a fertilization dish under mineral oil and cumulus–oocyte complexes (COCs) were transferred into the drop of fertilization medium. Twenty to 30 COCs were incubated in each drop for at least 0.5 h until insemination. A frozen microtube containing a sperm suspension frozen by the EQ method was removed from a deep freezer or LN_2_, kept in air at room temperature for 10 s, and immersed in warmed water at 37 °C for 15 min (Fig. 2B). During warming, the tops of the microtubes were kept above the water using a floating foam tube rack (Fig. 2B). When microtubes had been kept in a LN_2_ tank, any LN_2_ inside the tube needed to be decanted before warming. After warming, 23–25 μL of the sperm suspension was transferred to a 300-μL droplet of preincubation medium (Fig. 2B). After 40–50 min, 15-μL aliquots of the sperm suspension were aspirated from the periphery of the drop and transferred into 80-μL drops of IVF medium containing COCs (Fig. 2B). At 3–4 h after insemination, morphologically normal oocytes were transferred into drops of CZB medium^19^ containing 5.6 mM glucose, 0.1 mg/mL PVA, and 3.0 mg/mL BSA and cultured at 37 °C under 5% CO_2_ in humid air for approximately 24 h. The next day, oocytes that had developed into normal-appearing 2-cell embryos were considered to have been fertilized. For thawing spermatozoa frozen by the standard method, the straws were removed from LN_2_, exposed to room temperature for 10 s, and then immersed in a water bath at 37 °C for 15 min. After warming, the sperm solution was expelled from the straw onto the bottom of a plastic dish using a metal stick. An aliquot of 5 μL thawed sperm suspension was taken from the periphery of the drop and added to 200 μL of HTF medium for preincubation and used for IVF as described above.

### Embryo transfer

Two-cell embryos were transferred into oviducts of both sides of day 1 pseudopregnant females of the ICR strain. Recipient females were each injected subcutaneously with 2 mg progesterone (Progehormone, Mochida Pharmaceutical Co. Ltd, Tokyo, Japan) in the evening on days 18 and 19 to prevent natural parturition. On day 20 in the morning (09:00 to 12:00), the recipient females were examined for the presence of fetuses by Cesarian section and live pups were then nursed by lactating ICR strain foster mothers.

### Assessment of sperm plasma membrane integrity and acrosomal status

Spermatozoa were subjected to propidium iodide (PI) and peanut agglutinin (PNA) staining to confirm the integrity of the plasma membrane and acrosomal status, respectively^7,20^. Briefly, after preincubation of thawed spermatozoa in HTF for 1 h, a 20-μL aliquot of suspension was placed in a tube and stained with 1 μL of PNA conjugated with Alexa Fluor 488 (1 mg/mL, Invitrogen, Paisley, UK) mixed with 0.5 μL of PI (2.4 mM, LIVE/DEAD Sperm Viability Kit; Molecular Probes, Eugene, OR, USA) without fixation. The tube was incubated in a dark box at 37 °C for 15 min. Two hundred spermatozoa per sample were observed under a fluorescent microscope (n = 3 replicates per group). In the results, PI ( +) indicates spermatozoa with damaged membrane and PNA ( +) represents spermatozoa with damaged acrosome or in the middle of the acrosomal reaction; the latter state occurs when PNA reaches the acrosomal contents through pores in the outer acrosomal membrane and plasmalemma during the acrosome reaction.

### Assessment of DNA damage status

Terminal deoxynucleotidyl transferase-mediated dUTP nick-end labeling (TUNEL) staining of spermatozoa was performed to assess DNA damage using In Situ Cell Death Detection kits, Fluorescein (Roche Applied Science, Mannheim, Germany)^21,22^. Briefly, spermatozoa were fixed in 3.6% paraformaldehyde (PFA) at room temperature for 30 min. After centrifugation (2,000 g, 5 min) and washing, fixed spermatozoa were permeabilized with 0.1% Triton X-100 containing 0.1% sodium citrate for 4 min in ice. Spermatozoa were washed twice by centrifugation, and incubated in 50 µL of labeling mixture for 1 h at 37 °C. Finally, spermatozoa were washed twice by centrifugation, and then mounted on slides. Signal intensities of TUNEL staining were detected using a C2 confocal microscope (NIKON, Tokyo, Japan), and quantified using ImageJ software (version 1.53, NIH, Bethesda, MD; https://imagej.nih.gov/ij/download.html). For all images of TUNEL staining, the same linear adjustment of the contrast across the images was performed to assure data consistency.

### Assessment of oxidative stress

Spermatozoa were stained to assess oxidative stress by 30-min incubation (37 °C) in HTF containing 5 µM CellROX Deep Red (Thermo Fisher Scientific, Waltham, MA)^23^ and 5 µg/mL Hoechst 33,342 (FUJIFILM Wako Pure Chemical Corporation, Osaka, Japan). As a positive control, 0.2 mM of hydrogen peroxide was added to fresh sperm solutions and tested in the same way. After centrifugation (2,000 g, 5 min), the supernatant was removed and spermatozoa were fixed with 4% PFA for 15 min. The suspension was recentrifuged and resuspended in 20 µL PBS. Then, 4 µL of sperm suspension was put on a glass slide and covered with a cover slip. Observation was performed using a C2 confocal microscope. CellROX intensity in the midpiece of spermatozoa was quantified by ImageJ software (see above).

### Statistical analysis

The percentages of motile spermatozoa, as well as the values of the kinetic parameters of spermatozoa (VAP, VSL, VCL, ALH, BCF, LIN, and STR), were analyzed by one-way analysis of variance (ANOVA) using Excel software (Statcel v. 2; Microsoft Corp., Edmond, WA, USA). Data calculated as percentages were subjected to arcsine transformation before performing ANOVA. The Tukey–Kramer test was used for multiple comparisons. Fertilization rates were analyzed using Mann–Whitney nonparametric *U* tests. Implantation and birth rates were analyzed using Fisher’s exact probability test; *P* < 0.05 was considered statistically significant.

## Results

### Cooling rates during freezing

**Figure 3:**
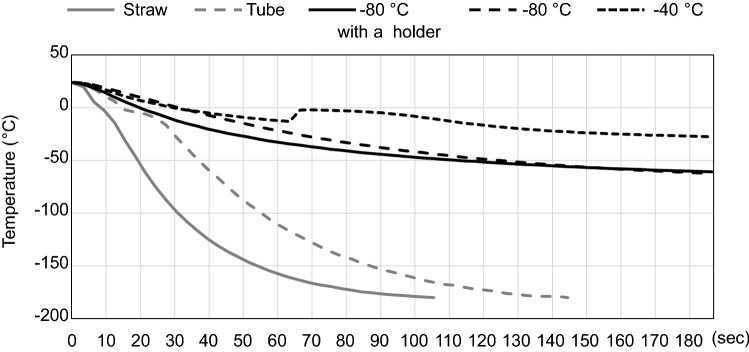
The cooling rates of mouse sperm suspensions frozen by various methods. Temperature changes were measured for the following conditions: the standard method using a plastic straw frozen in LN_2_ (gray, solid line); using a cryotube frozen in LN_2_^12^ (gray, dotted line); the EQ method using a microtube frozen in a −80 °C freezer (black, solid line) or in a −40 °C freezer (black, fine dotted line). The EQ method without an aluminum foil stand was also tested (black, coarse dotted line).

First, we analyzed the cooling rates of spermatozoa frozen by three different methods: the standard method using plastic straws, a previously developed cryotube method^12^, or by our EQ method. Plastic straws and cryotubes were frozen in the gas and liquid phases of LN_2_, respectively. Microtubes in the EQ method were frozen at −80 °C or −40 °C with or without a precooled aluminum foil stand. The standard method gave the most rapid cooling rate, attaining –100 °C within about 30 s (Fig. 3). With a cryotube cooled in the liquid phase of LN_2_, the cooling rate was moderate, reaching −25 °C at 30 s. It is important to note that the EQ method, despite the use of a −80 °C freezer, attained a cooling rate similar to that of the cryotube method during the first 30 s (Fig. 3). However, when cooled without a precooled aluminum foil stand or cooled at −40 °C, the cooling rate slowed and the temperature never reached below 0 °C by 30 s (Fig. 3). Based on these results, we employed the protocol using aluminum foil stands and a −80 °C freezer for all the IVF experiments described below.

### Optimization of sperm freezing solution for the EQ method

Next, we sought to optimize the raffinose concentration for the EQ method. When spermatozoa were frozen in solutions containing 9% to 21% raffinose in DW, the motility and progressive motility of the thawed spermatozoa increased in a concentration-dependent manner (Supplementary Fig. S1A), but sperm velocity did not change significantly (Supplementary Fig. S1B). The degree of damage to the plasma membrane assessed by PI staining showed a trend toward improvement in 18% and 21% raffinose solutions (Supplementary Fig. S1C). Damage to the acrosomal membrane identified by PNA staining tended to be higher in the 21% raffinose solution (Supplementary Fig. S1C). We then assessed the combinations of different raffinose solutions (15%, 18%, and 21%) and different solvents (DW or SD-PBS with dilutions from 1/4 to 3/4) for sperm freezing. After thawing, the highest sperm motility was obtained with 18% or 21% raffinose in 1/4 SD-PBS solution (Supplementary Fig. S1D). However, these sperm motilities were not significantly different from those frozen in 18–21% raffinose in DW. Based on these results, we decided to use 20% raffinose solution in DW for the EQ method to enable easier preparation of the freezing solution.

### Kinetic analysis of frozen–thawed spermatozoa

**Figure 4:**
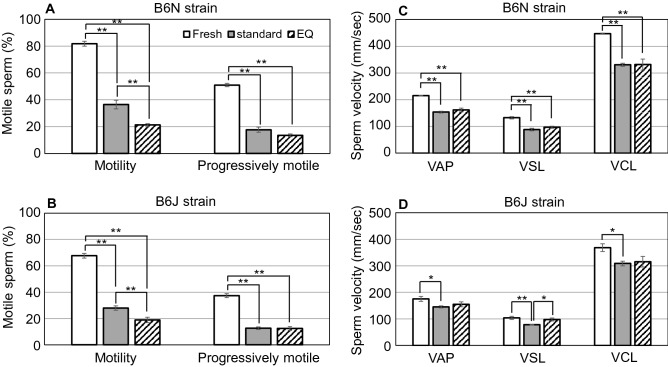
Results of sperm kinetic analysis. The motility rate, progressive motility rate and three kinds of velocity parameters were measured using frozen–thawed spermatozoa from B6N (n = 6) (**A,C**) and B6J (n = 7) (**B,D**) mouse strains. The asterisks indicate significant differences between groups (*P* < 0.05 by Tukey–Kramer test).

The percentages of motile spermatozoa after freezing at −80 °C by the EQ method were lower than those of the standard method for both the B6N and B6J strains (Fig. 4). However, the progressive motility and three of the sperm velocity parameters were not significantly different between the two methods, except for VSL in the B6J group (Fig. 4). Spermatozoa frozen with the EQ method showed significantly higher linear motility parameters (LIN and STR) than those from fresh preparations and following the standard freezing method (Supplementary Table S1). This was more evident in the B6J than in the B6N strain, probably reflecting the lower ALH (amplitude of lateral head movement) in the former (Supplementary Table S1). This difference in the linearity of sperm movement might be associated with the difference in IVF rates between these substrains. We also analyzed B6J spermatozoa frozen at −40 °C by the EQ method, but motility was less than 1% in all five samples, probably reflecting the slow cooling rate above.

### Sperm freezing and IVF using frozen–thawed spermatozoa

**Figure 5:**
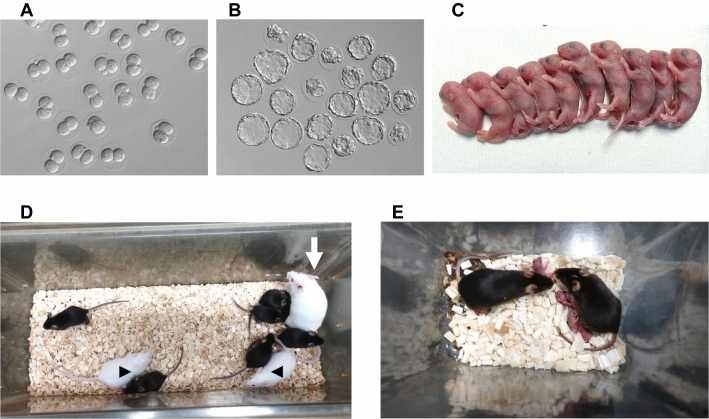
Confirmation of developmental abilities after IVF using frozen–thawed spermatozoa cryopreserved using the EQ method. (**A**) Two-cell stage embryos at 24 h after insemination. (**B**) Blastocysts after in vitro culture for 120 h. (**C**) A litter born by Cesarian section from the transfer of 2-cell stage embryos. (**D**) Pups (black hair color) survived to weaning and the figure shows their foster mother (arrow) and mother’s pups (arrowheads). (**E**) Newborn pups from the mating of pairs of mice derived from frozen–thawed spermatozoa.

In the first series of IVF experiments, we used spermatozoa from B6N and B6J strain mice frozen by the EQ method and stored at −80 °C for 1 month. The fertilization rates after conventional IVF were 50% and 31%, respectively, which were significantly lower (*P* < 0.05) than those of IVF using spermatozoa frozen by the standard method (Table 1). To improve the fertilization rate when using spermatozoa frozen by the EQ method, the Ca^2+^ concentration in the preincubation medium was increased to 5 mM. The fertilization rates increased in both strain groups, reaching 57% and 51%, respectively (Table 1). The 2-cell embryos thus obtained, and the blastocysts obtained following in vitro culture (23/30 2-cells; 77%) were morphologically normal (Fig. 5A, B). To confirm the in vivo developmental potential of these 2-cell stage embryos, they were transferred into the oviducts of pseudopregnant female mice. As a result, 87% and 82% of them implanted and 58% and 55% developed into offspring, respectively (Table 2, Fig. 5C). Thirteen of 14 offspring tested survived to weaning (Fig. 5D). We confirmed the fertility of all male offspring (n = 6) by the formation of a vaginal plug in females at mating and birth of normal offspring (Fig. 5E). Thus, spermatozoa frozen by the EQ method had adequate fertilizing ability in vitro, producing normal embryos and offspring at practicable efficiencies for preserving valuable strains.

**Table 1:** Fertilization rates of oocytes following IVF using spermatozoa frozen by the standard or EQ methods.

Strain of males	Method	Ca^2+^ concentration (mM)	No. of epididymides used	Total no. (%) of oocytes fertilized/inseminated	Mean % of fertilization (± SEM)
B6N	Standard	2	8	427/469 (91.0)	91.4 (± 1.5)
	EQ	2	7	209/401 (52.1)	49.9 (± 7.0)**
		5	7	176/307 (57.3)	56.7 (± 4.9)**
B6J	Standard	2	10	583/754 (77.3)	77.3 (± 1.4)
	EQ	2	7	118/380 (31.1)	30.8 (± 2.0)**
		5	7	150/301 (49.3)	50.9 (± 10.5)*

**P < 0.01, *P < 0.05 vs standard cryopreservation method for the same strain by Mann–Whitney nonparametric *U* test. SEM, standard error of the mean.

**Table 2:** Full-term development of embryos fertilized in vitro using spermatozoa frozen by the EQ method and stored for 1 month.

Strain of males	No. (%) of females used			No. (%) of embryos				
	Transferred	Pregnant	(%)	Transferred	Implanted	(%)	Developed to offspring	(%)
B6N	3	3	(100)	45	39	(87)	26	(58)
B6J	4	4	(100)	60	49	(82)	33	(55)

There were no significant differences in the implantation and birth rates between strains using Fisher’s exact test.

We also asked three individuals with no experience of sperm freezing to freeze mouse spermatozoa according to our EQ protocol. Following IVF using frozen–thawed spermatozoa, 63% (32/51), 69% (42/61), and 74% (52/70), respectively, of oocytes were fertilized (Supplementary Table S2). Thus, our EQ method is reproducible, even by such inexperienced persons.

### Extending the sperm storage period and storage in LN_2_

We next examined whether the fertilizing ability of spermatozoa frozen by the EQ method was affected by extended storage. In all experiments, frozen–thawed B6J spermatozoa preincubated in the presence of 5 mM Ca^2+^ were used for IVF. Even when spermatozoa were stored at −80 °C for 3 months, oocytes were fertilized in all replicate experiments (*n* = 5) and the mean fertilization rate was 24.9% (Table 3). Of the fertilized oocytes, 25/40 (63%) developed into term offspring following embryo transfer to pseudopregnant recipient females (Table 4). When spermatozoa were stored at –80 °C for 1 month followed by storage in LN_2_ for 2 months, fertilized oocytes were obtained in all replicates and a mean fertilization rate of 28.6% was obtained (Table 3). Of these embryos, 21/38 (55%) developed into offspring after transfer (Table 4). These implantation rates and birth rates were not significantly different from those obtained using spermatozoa frozen by the standard method, although the fertilization rates were significantly lower (Tables 3 and 4). Finally, we found that, even after storage at −80 °C for 9 months, they still maintained a practical fertilization rate of 33.4 ± 9.2% (n = 5).

**Table 3:** Fertilization rates following IVF using B6J sperm frozen by the standard method and stored for 2.5 years and the EQ method and stored for 1–3 months.

Method	Storage term at		No. of epididymides used	Total no. (%) of oocytes fertilized/ inseminated	Mean % of fertilization (± SEM)
	−80 °C	−196 °C			
Standard	–	2.5 years	6	177/199 (89.0)	89.0 (± 3.8)
EQ	3 months	–	5	41/165 (24.8)	24.9 (± 4.5)**
EQ	1 month	2 months	5	47/171 (27.5)	28.6 (± 4.9)**

**P < 0.01 vs standard cryopreservation method by Mann–Whitney nonparametric *U* test. SEM, standard error of the mean.

**Table 4:** Full-term development of embryos fertilized in vitro using spermatozoa frozen by the standard method and stored for 2.5 years or by the EQ method and stored for 1–3 months.

Method	Storage term at		No. (%) of females			No. (%) of embryos				
	−80 °C	−196 °C	Transferred	Pregnant	(%)	Transferred	Implanted	(%)	Developed to offspring	(%)
Standard	–	2.5 years	10	10	(100)	156	123	(79)	91	(58)
EQ	3 months	–	3	3	(100)	40	26	(65)	25	(63)
EQ	1 month	2 months	3	3	(100)	38	26	(68)	21	(55)

There were no significant differences in the implantation or birth rates between the groups by Fisher’s exact test.

### Evaluation of damage to spermatozoa frozen by different methods

**Figure 6:**
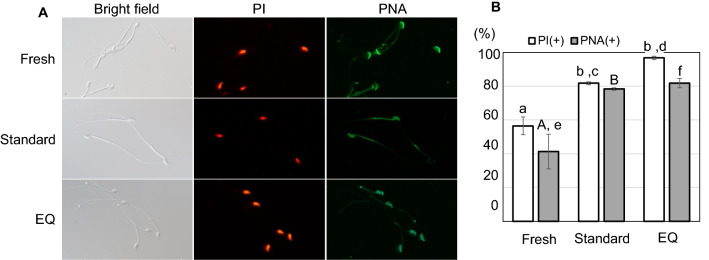
Visualization of plasma membrane integrity and the acrosomal status. (**A**) Representative images of fresh and frozen–thawed spermatozoa by standard and EQ methods after staining using propidium iodide (PI; red) and peanut agglutinin (PNA; green). (**B**) The percentages of spermatozoa stained by PI ( +) and PNA ( +). Significant difference indicated by different superscript letters. (^a, b; c, d; e, f^ *P* < 0.01, ^A, B^ *P* < 0.05 by Tukey–Kramer test).

**Figure 7:**
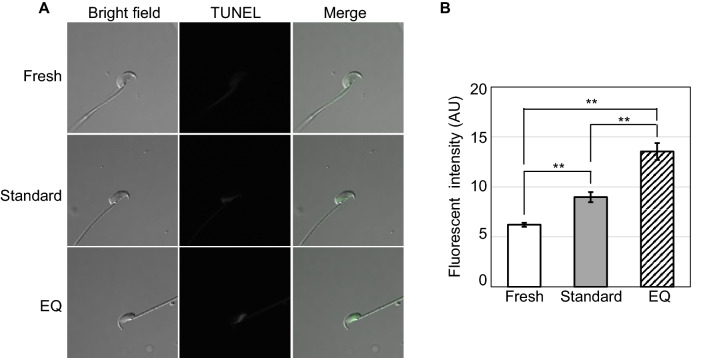
Visualization of DNA damage status by TUNEL assay. (**A**) Representative images of fresh and frozen–thawed spermatozoa cryopreserved by standard or EQ methods after TUNEL staining. (**B**) The relative fluorescent intensities of TUNEL signals (fresh, n = 93; standard method, n = 96; EQ, n = 83). The asterisks indicate significant differences between groups (**P < 0.01 by Tukey–Kramer test).

**Figure 8:**
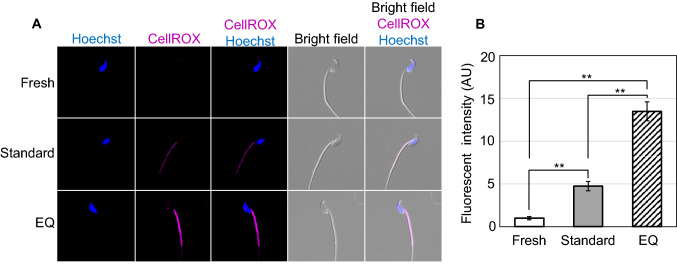
Visualization of oxidative stress by CellROX Deep Red staining. (**A**) Representative images of fresh and frozen–thawed spermatozoa cryopreserved by standard or EQ methods after staining with Hoechst 33,342 and CellROX Deep Red. (**B**) Relative fluorescent intensities of CellROX Deep Red signals. **P < 0.01 between groups by Tukey–Kramer test.

As shown above, the fertilizing ability of spermatozoa frozen by the EQ method was lower than those frozen by the standard method or with fresh spermatozoa. We then examined how the quality of spermatozoa was affected by the EQ method using different assessment protocols. PI staining for plasma membrane integrity showed that 97% of spermatozoa frozen by the EQ method were damaged, significantly higher than that of spermatozoa frozen–thawed by the standard method (82%) or fresh spermatozoa (57%) (Fig. 6). The acrosomal status assessed by PNA staining showed significant damage among spermatozoa frozen by the EQ and standard methods, although there was no significant difference between them (Fig. 6). PNA staining normally demonstrated intense florescence at the acrosomal region of about 40% of fresh spermatozoa, whereas the staining was broad and moderate over the entire area of the head in about 80% of spermatozoa frozen by either method (Fig. 6) for unknown reasons^7^. TUNEL assays for DNA damage revealed positive signals around the postacrosomal region of the sperm head. Sperm freezing significantly increased the signal intensity compared with fresh control, with the highest signal intensity seen following the EQ method group (Fig. 7). To evaluate oxidative stress, spermatozoa were stained with CellROX Deep Red. Oxidative stress signals were detected in the midpiece regions of spermatozoa (Fig. 8). Higher signals were detected in spermatozoa frozen by either method than in fresh spermatozoa, with the highest signal intensity in the EQ group (Fig. 8).

## Discussion

As far as we know, the EQ method we report here is the easiest and quickest method for mouse sperm freezing among the existing protocols that can still maintain an adequate fertilizing ability of frozen–thawed spermatozoa by IVF. The EQ method does not require special storage containers (e.g., plastic straws), chemically undefined reagents (e.g., skim milk), or access to LN_2_. More importantly, it does not require preparation or handling of sperm suspensions, which ensures reproducibility of the results even by persons who have no experience of sperm freezing. For successful sperm freezing by this EQ method, it is necessary to achieve sufficient cooling rates in a freezer using precooled aluminum foil stands and a deep freezer at −80 °C, not −40 °C. The cooling rate for the first 30 s by the EQ method was slower than that of the standard method using plastic straws and LN_2_, but similar to that of the cryotube method using LN_2_. During this period, the sperm suspension reached −12 °C, at which point the sperm suspension might have shifted from the liquid to the solid (frozen) phase. This phase shift should probably proceed rapidly enough to avoid cryodamage to the spermatozoa^24^, and plastic straws might be better for this. Indeed, it was reported that mouse spermatozoa could be frozen and stored in plastic straws at −80 °C for 1 year, but this required preparation of a sperm suspension and a 3% skim milk/18% raffinose solution^25^. Alternatively, the efficiency of the EQ method can be improved if LN_2_ is available such that the microtubes can be frozen by placing them up to 1 cm below the LN_2_ surface, as we have shown previously^12^.

In the EQ method, a simple 20% raffinose solution was employed based on our detailed factorial analysis (Supplementary Fig. S1) and used instead of the standard 3% skim milk/18% raffinose solution. According to our previous measurements, the osmolality of the 20% raffinose solution is approximately 400 mOsmol/kg, which is within the range of optimal osmolality for sperm freezing (350–600 mOsmol/kg)^26,27^. Preparation of the standard raffinose solution requires removal of sediments by three rounds of centrifugation at 17,000 g for 15 min in our laboratory. By contrast, the EQ method does not require centrifugation and the solution can be prepared easily. Furthermore, it is desirable to avoid the use of chemically undefined skim milk if a retrospective IVF test is not performed.

Another advantage of the EQ method is its speed and ease of use. In our handling, the typical time for sperm freezing using three male mice was about 7–8 min, which is less than one-third of the time required for the standard method. This is because the main process for the preparation of a sperm suspension and placing it into the freezing container involves a single step. By the standard method, sperm suspensions should be prepared by allowing spermatozoa to disperse in medium on a culture dish, and these are then placed carefully in plastic straws with appropriate sealing of both ends. These processes are not always technically difficult but require some practice. By contrast, we show here that the EQ method can be performed by persons without any experience of sperm freezing.

The EQ method has two major drawbacks. One is the lower fertilization rates by IVF compared with those of the standard methods. However, we obtained a > 50% fertilization rate even with 1-month storage of sperm suspensions from B6J mice, which are known to be highly sensitive to freezing. This fertilization rate of ~ 50% is sufficient in practice because oocytes can be prepared from wild-type females in most cases. We found that increasing the Ca^2+^ concentration in the preincubation medium effectively improved the fertilization rates. This adjustment was proven to promote sperm capacitation^28,29^ and to improve the fertilization rate^30^. Intriguingly, the rate of progressive motility and the velocity parameters in frozen–thawed spermatozoa were mostly similar between the EQ method and the standard freeze–thawing method. Furthermore, the linear movement characteristics (LIN and STR) of spermatozoa frozen by the EQ method were higher than those frozen by the standard method. The degrees of oxidative stress and damage of the plasma membrane and the acrosome were higher with the EQ method than the standard method. We assume that the cumulative effects of all these factors resulted in the low, but still practical, fertilization rates with the EQ method. It may be possible to increase the efficiency of the EQ method by improving these parameters. Another drawback of the EQ method is the limited storage time at −80 °C, but we confirmed that 9 months of storage was possible. It is expected that, during this period, those mouse strains cryopreserved by the EQ method could be revived by IVF and embryo transfer, or by transfer to LN_2_ at −196 °C for permanent storage.

In conclusion, we consider that the EQ method can be reliably used for temporary strain preservation in the event of a possible emergency suspension of animal care. An accidental microbiological contamination can occur at any time in any animal facility. Furthermore, serious social emergencies might not allow routine animal care, as with the COVID-19 pandemic that started in early 2020. The strains may be revived safely after a few months of storage in −80 °C freezers or after a longer storage period by transferring the spermatozoa into LN_2_. If LN_2_ is not available to the animal facility, the frozen spermatozoa can be transported at −80 °C using dry ice to other places where they can be stored permanently in LN_2_. The various mouse repository centers around the world, including ours, might be practical choices for this purpose.

## Supplementary Information

Supplementary Information.

## Data Availability

All data are included in this paper or the Supplementary Materials.
