# Supplementary material for: Easy and quick (EQ) sperm freezing method for urgent preservation of mouse strains
Source: Sci Rep. 2021 Jul 8;11:14149. doi: 10.1038/s41598-021-93604-y (PMC8266870; doi:10.1038/s41598-021-93604-y)
Supplement: Supplementary file 1 — Supplementary Information. [file 41598_2021_93604_MOESM1_ESM.docx]

Supplementary material

**Easy and quick (EQ) sperm freezing method for urgent preservation of mouse strains**

Keiji Mochida^1,5 *^, Ayumi Hasegawa^1,5^, Daiki Shikata^1^, Nobuhiko Itami^1^, Masashi Hada^1^, Naomi Watanabe^1,2^, Toshiko Tomishima^1^, Atsuo Ogura^1,2,3,4*^

^1^RIKEN BioResouce Research Center, Tsukuba, Ibaraki 305-0074, Japan, ^2^Graduate School of Life and Environmental Science, University of Tsukuba, Tsukuba, Ibaraki 305- 8572, Japan, ^3^Center for Disease Biology and Integrative Medicine, Faculty of Medicine, University of Tokyo, Bunkyo-ku, Tokyo, Japan, and ^4^RIKEN Cluster for Pioneering Research, Hirosawa, Wako, Saitama 351-0198, Japan

^5^These authors contributed equally: Keiji Mochida and Ayumi Hasegawa


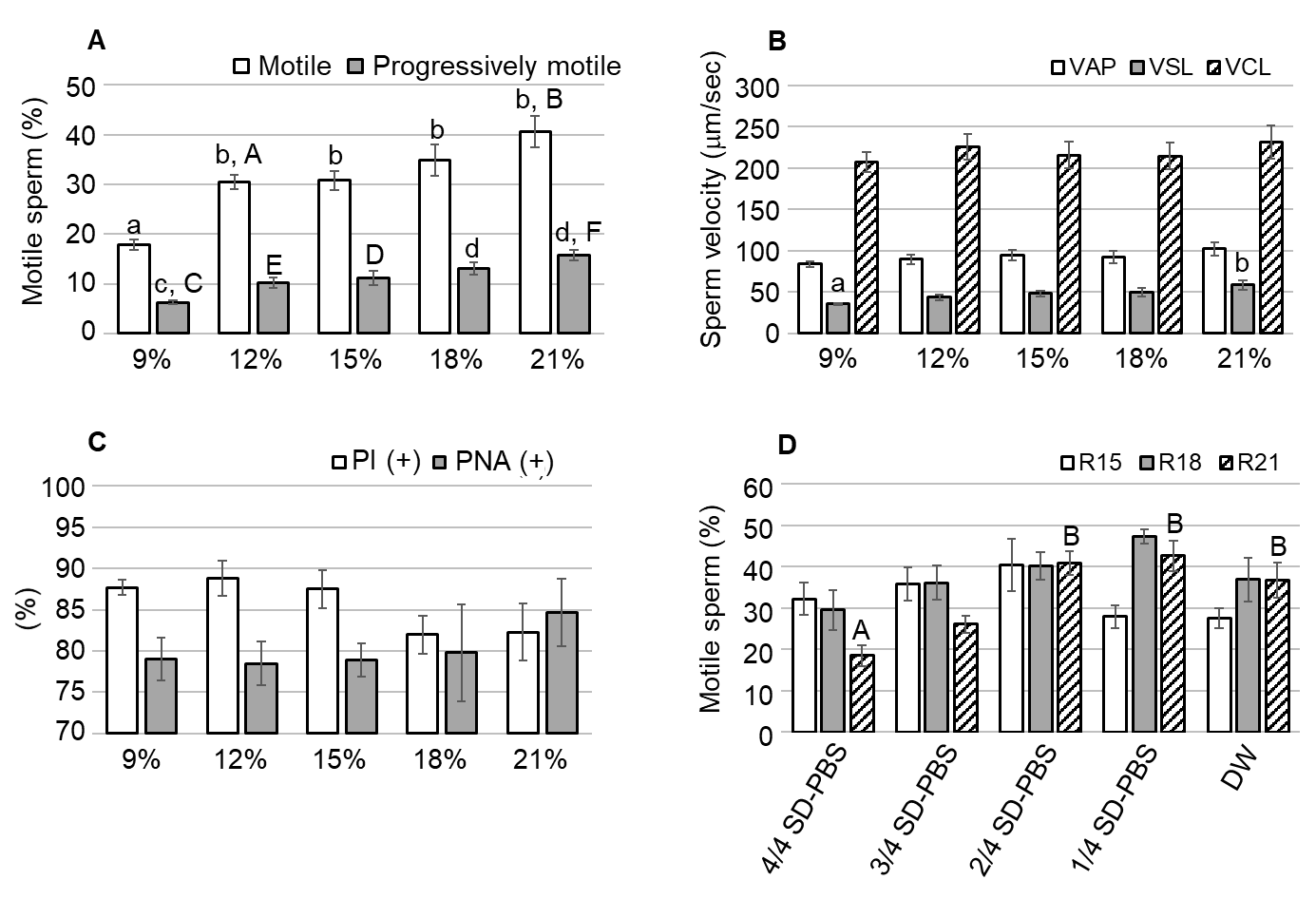


**Figure S1. Examination of conditions for suitable mouse sperm cryopreservation solution.** The effect of raffinose concentration and motility (A), velocity parameters (B), damage rates (C) of plasma membrane (PI staining) and acrosome (PNA staining), and solvent concentration and motility (D) are shown. Different superscript letters indicate significant differences between groups (^a, b; c, d^ *P* < 0.01, ^A, B; C, D; E, F^ *P* < 0.05 by Tukey–Kramer test).

**Table S1. Results of sperm kinetic analysis for nine motility parameters.**

| Strain | Method | Motility (%) | Progressively motile (%) | Velocity (mm/s) | | | ALH (mm) | BCF (Hz) | LIN (%) | STR (%) |
| --- | --- | --- | --- | --- | --- | --- | --- | --- | --- | --- |
|  |  |  |  | VAP | VSL | VCL |  |  |  |  |
| B6N | Fresh | 81.8^a^ | 51.0^a^ | 215.3^a^ | 132.5^a^ | 447.5^a^ | 27.6^a^ | 34.5 | 28.7^A^ | 59.0 |
|  | Standard | 36.5^b^ | 17.7^b^ | 153.7^b^ | 88.2^b^ | 331.0^b^ | 22.6^b^ | 37.4 | 28.5^A^ | 59.2 |
|  | EQ | 21.2^c^ | 13.5^b^ | 161.4^b^ | 96.7^b^ | 331.9^b^ | 22.5^b^ | 37.5 | 31.7^B^ | 61.8 |
| B6J | Fresh | 67.7^a^ | 37.3^a^ | 175.1^A^ | 104.1^a^ | 368.4^A^ | 23.8^A^ | 34.7 | 28.9^A^ | 59.4^A^ |
|  | Standard | 28.0^b^ | 12.7^b^ | 145.4^B^ | 78.4^b,A^ | 309.2^B^ | 22.6^A^ | 36.7 | 27.6^A^ | 57.3^A^ |
|  | EQ | 18.9^c^ | 12.4^b^ | 155.0 | 97.5^B^ | 315.6 | 21.1^B^ | 37.7 | 32.9^B^ | 64.7^B^ |

All parameters were compared between fresh and frozen–thawed sperm suspensions cryopreserved by standard and EQ methods in B6N and B6J strains. The means are from 6–7 male mice, The analysis was done within each strain, and columns and results with different superscript letters are significantly different: ^a,b; b,c; a,c^ *P* < 0.01; ^A,B^ *P* < 0.05 by Tukey–Kramer test. Motility parameters from computer-assisted sperm movement analysis are: VAP, average path velocity; VSL, straight-line velocity; VCL, curvilinear velocity; ALH, amplitude of lateral head displacement; BCF, beat cross frequency; LIN, linearity; STR, straightness.

| **Table S2. Fertilization rates following IVF using spermatozoa frozen by inexperienced persons with the EQ method.** | | | |
| --- | --- | --- | --- |
|  | Person #1 | Person #2 | Person #3 |
| 1st IVF | 9/17 | 9/18 | 52/70 |
| 2nd IVF | 23/34 | 33/43 | not done |
| Total | 32/51 (63%) | 42/61 (69%) | 52/70 (74%) |
| B6J spermatozoa frozen by the EQ method were stored at –80 °C for 1 to 2 weeks before thawing for IVF experiments. | | | |
